# Healthcare-associated infections and antimicrobial use in acute care hospitals: a point prevalence survey in Lombardy, Italy, in 2022

**DOI:** 10.1186/s12879-024-09487-7

**Published:** 2024-06-25

**Authors:** Antonio Antonelli, Maria Elena Ales, Greta Chiecca, Zeno Dalla Valle, Emanuele De Ponti, Danilo Cereda, Lucia Crottogini, Cristina Renzi, Carlo Signorelli, Matteo Moro

**Affiliations:** 1 Università Vita Salute San Raffaele, Via Olgettina 58, Milan, 20132 Italy; 2 Regione Lombardia Direzione Generale Welfare, Milan, Italy; 3 IRCCS Ospedale San Raffaele, Milan, Italy; 4 Epidemiology of Cancer Healthcare & Outcomes (ECHO) Research Group, Department of Behavioural Science and Health, Institute of Epidemiology & Health Care, University College London, WC1E 7HB, London, UK

**Keywords:** Point prevalence survey, Healthcare associated infection, Antimicrobial use, Antimicrobial stewardship, Surveillance, Infection prevention and control

## Abstract

**Background:**

Healthcare-Associated Infections (HAIs) are a global public health issue, representing a significant burden of disease that leads to prolonged hospital stays, inappropriate use of antimicrobial drugs, intricately linked to the development of resistant microorganisms, and higher costs for healthcare systems. The study aimed to measure the prevalence of HAIs, the use of antimicrobials, and assess healthcare- and patient-related risk factors, to help identify key intervention points for effectively reducing the burden of HAIs.

**Methods:**

A total of 28 acute care hospitals in the Lombardy region, Northern Italy, participated in the third European Point Prevalence Survey (PPS-3) coordinated by ECDC for the surveillance of HAIs in acute care hospitals (Protocol 6.0).

**Results:**

HAIs were detected in 1,259 (10.1%, 95% CI 9.6–10.7%) out of 12,412 enrolled patients. 1,385 HAIs were reported (1.1 HAIs per patient on average). The most common types of HAIs were bloodstream infections (262 cases, 18.9%), urinary tract infections (237, 17.1%), SARS-CoV-2 infections (236, 17.0%), pneumonia and lower respiratory tract infections (231, 16.7%), and surgical site infections (152, 11.0%). Excluding SARS-CoV-2 infections, the overall prevalence of HAIs was 8.4% (95% CI 7.9–8.9%). HAIs were significantly more frequent in patients hospitalized in smaller hospitals and in intensive care units (ICUs), among males, advanced age, severe clinical condition and in patients using invasive medical devices. Overall, 5,225 patients (42.1%, 95% CI 41.3–43.0%) received systemic antimicrobial therapy. According to the WHO’s AWaRe classification, the Access group accounted for 32.7% of total antibiotic consumption, while Watch and Reserve classes accounted for 57.0% and 5.9% respectively. From a microbiological perspective, investigations were conducted on only 64% of the HAIs, showing, however, a significant pattern of antibiotic resistance.

**Conclusions:**

The PPS-3 in Lombardy, involving data collection on HAIs and antimicrobial use in acute care hospitals, highlights the crucial need for a structured framework serving both as a valuable benchmark for individual hospitals and as a foundation to effectively channel interventions to the most critical areas, prioritizing future regional health policies to reduce the burden of HAIs.

**Supplementary Information:**

The online version contains supplementary material available at 10.1186/s12879-024-09487-7.

## Introduction

Healthcare-Associated Infections (HAIs) are a global public health issue, representing a significant burden of disease, suffering and mortality [1]. HAIs may lead to prolonged hospital stays [2], resulting in considerable costs for healthcare systems [3–5]; additionally, they are associated with an increased risk of inappropriate use of antimicrobial drugs and the development of resistant microorganisms [6], against which there will progressively be fewer and less effective antimicrobial drugs [7].

Literature highlights compelling evidence that the burden of HAIs can be mitigated through appropriate interventions [8]. However, despite the efforts [9], according to the most recent estimates from the second European Point Prevalence Survey held between 2016 and 2017, in Europe 8.9 million HAI occurred and 3.8 million patients experienced at least one HAI [10].

The surveillance of HAIs and of antimicrobial use is essential at hospital, regional, national, and international levels for providing a structured benchmarking framework and for informing appropriate and coordinated health policies [11–13]. The ECDC Point Prevalence Survey of healthcare-associated infections and antimicrobial use in European acute care hospitals (PPS) is a standardized data collection framework conducted every five years in the 27 EU/EEA countries, plus the UK and Serbia. The first PPS was carried out between 2011 and 2012 [14, 15], the second (PPS-2) between 2016 and 2017 [16] and the third and most recent one (PPS-3) between 2022 and 2023 [17].

In Italy, PPS-3 was nationally coordinated by the University of Turin, with data collection decentralized in each of the participating regions [18]. In Lombardy, the most densely populated region in Italy, 28 Acute Care Hospitals (ACHs) voluntarily participated. This study aims to examine the PPS-3 data collected in the Lombardy region between November and December 2022. In particular, we evaluated the prevalence of HAIs, the use of antimicrobials, as well as assessing healthcare-related factors and patient characteristics.

## Methods

### Study design and data collection

The survey was conducted following the ECDC Protocol 6.0 [17]. Each participating ACH submitted all the data regarding the hospital organization, the use of antimicrobials and the HAIs to a national data repository named RedCap [19, 20].

Data collection took place between November 3 and December 20, 2022, with each ward conducting data collection on a specific day. Access to the RedCap platform was granted to data entry operators following a training session on GDPR and data protection. This platform was also accessible to regional coordinators from the Welfare General Directorate of Lombardy Region, enabling them to access data for all hospitals in the region.

According to the protocol [17], all patients in the eligible wards were included and both hospital and patient data were anonymized during analysis.

ACHs were classified based on capacity in small (≤ 200), medium-size (201–499) and large (≥ 500 beds). Data were collected from the wards for each eligible patient, encompassing risk factors, the presence of HAIs and the use of at least one antimicrobic (grouped using the WHO AWaRe classification [21], when applicable); the protocol defines HAIs as active if symptoms occur on day 3 or later of the current admission, with specific exceptions regarding the timeframe to be considered in case of surgical site infections (SSI), infections related to an invasive medical device, *C. difficile* infections, and if the patient has been readmitted within 48 h.

### Statistical analysis

Initially, a coherence analysis was performed to identify records with logical inconsistencies resulting from errors during the form submission. A total of 14 flags were identified, and corrective actions were taken for each (details in the Supplementary Table 1).

Descriptive analyses included the median and Interquartile range (IQR) for continuous variables, and frequency distribution of categorical variables. The prevalence of HAIs, computed as the proportion of patients with at least one HAI, and antimicrobial use were stratified by epidemiologically significant variables according to previous ECDC report [16]. Confidence intervals were computed using the Clopper-Pearson exact method for proportions. We employed chi-square tests for evaluating whether the prevalence of HAIs and antibiotic use differed by healthcare- and patient-related factors.

Data on pan-drug resistant microorganism were cross verified with data available in the regional microbiological surveillance system for confirmation.

Data were analyzed using STATA version 18.0 (StataCorp. 2023. Stata Statistical Software: Release 18 College Station, TX: StataCorp LLC) and Python version 3.10.9 with the pandas library version 1.5.3.

## Results

Data were collected from 12,412 patients across 28 ACHs, comprising 39 acute care facilities throughout the Lombardy Region. Each hospital enrolled a median of 434 patients (IQR: 199–663). Participating facilities constituted 20% (39 out of 195) of all acute care facilities and accounted for 44% (18,620 out of 42,018) of acute care beds within the Region. Additionally, these facilities (5 small, 7 medium-sized, and 16 large hospitals) represented 50% (646,261 out of 1,288,198) of annual hospital admissions.

### Patients’ characteristics

Out of 12,412 patients enrolled in the study, 6,465 (52.2%) were male, 5,930 (47.8%) were female while the sex of 7 patients was unspecified. The median age was 68 years (IQR: 48–79, minimum 0, maximum 103). 740 patients were younger than 2 years, as infants were also included in the study.

Most enrolled patients were admitted to Medicine (14.7%), General Surgery (6.8%), and Cardiology (6.1%) wards. Other specialized wards each accounted for less than 5% of admissions.

Based on the estimated clinical severity assessed using the McCabe Score, 67.2% of the enrolled patients had a non-fatal disease (expected survival > 5 years), 16.6% had an ultimately fatal disease (expected survival 1 to 5 years), 6.6% had a rapidly fatal disease (expected survival < 1 year), and in 9.6% of cases the McCabe Score was unknown or unregistered.

Of the enrolled patients, 34% (4,278) underwent surgery on the day of the study, and among them 2,783 (22%) underwent major surgery, and 1,495 (12%) underwent a minimally invasive surgery.

### Use of invasive medical devices

4,548 (36.6%) patients had at least one invasive medical device (MD) in place (urinary catheter, central venous catheter, and/or intubation), specifically 3,468 (76.3%) had only one device, 766 (16.8%) had two, and 314 (6.9%) had three MDs.

The most used MD was the urinary catheter, 3,582 (29.1%) patients, followed by the central venous catheter (1,919, 15.5%) and intubation (441, 3.6%).

The number and type of MDs varied by care area, with the highest utilization observed in the intensive care units (71.9% of patients using at least one device), followed by medical (43.3%) and surgical (39.2%) wards.

### Prevalence of HAIs

Healthcare-associated infections were detected in 1,259 patients, resulting in a prevalence of 10.1% (95% CI 9.6–10.7%). In total, 1,385 HAIs were reported, with 1.1 HAIs per patient on average.

Among the participating ACHs, the prevalence of HAIs varied significantly, ranging from 1.4% (95% CI 0.2-5.0%) to 28.2% (95% CI 18.6–39.5%).

Specifically, 260 (2.1%) patients had at least one HAI upon hospital admission, while 998 (8.0%) patients developed the HAI during their stay in the hospital. Excluding hospital-acquired SARS-CoV-2 infections from the analysis, the overall prevalence of HAIs was 8.4% (95% CI 7.9–8.9%), affecting 1,045 patients. Of these, 795 (6.4%) developed at least one HAI during their hospitalization and 247 (2.0%) had an HAI present upon admission.

As shown in Table 1, the prevalence of HAIs was significantly higher in men, in patients aged over 64 years, in those with severe McCabe score or having undergone major surgery. When stratifying by hospital size, a higher prevalence of HAIs was observed in small and large hospitals compared to medium-sized hospitals. Further stratification by healthcare areas revealed an elevated prevalence within intensive care units (ICUs), excluding long-term care due to very limited data. Additionally, the presence of invasive medical devices was linked to a higher HAI prevalence, reaching 31.3% among intubated patients.

**Table 1:** Prevalence of HAIs stratified by main risk factors

Variable	TOTAL No.	% with HAI	95% CI	*P*-value
Overall	12,412	10.1	9.6–10.7	
Patient characteristics				
**Sex**				< 0.001
Male	6,465	11.5	10.7–12.3	
Female	5,930	8.6	7.9–9.4	
**Age class**				< 0.001
> 64	6,812	12.5	11.7–13.3	
15–64	4,370	8.0	7.2–8.9	
< 15	1,146	4.3	3.2–5.6	
**McCabe score**				< 0.001
Non-fatal	8,332	7.5	7.0-8.2	
Fatal	2,057	18.0	16.3–19.7	
Unknown	1,200	10.4	8.7–12.3	
Rapidly fatal	815	16.2	13.7–18.9	
**Medical Devices**				
Intubation				< 0.001
No	11,965	9.4	8.8–9.9	
Yes	441	31.1	26.8–35.6	
CVC				< 0.001
No	10,488	7.5	7.0–8.0	
Yes	1,919	24.8	22.9–26.8	
Urinary catheter				< 0.001
No	8,823	7.3	6.7–7.8	
Yes	3,582	17.2	16.0-18.5	
**Surgery**				< 0.001
No	8,057	8.6	7.8–9.2	
Major	2,783	14.4	13.1–15.8	
Minimally invasive	1,495	11.0	9.5–12.7	
Unknown	72	4.2	0.9–11.7	
ACHs characteristics				
**Hospital size (n. of beds)**				< 0.001
> 500	10,319	10.5	10.0-11.1	
201–500	1,793	7.4	6.2–8.7	
< 200	283	13.8	10.0-18.3	
**Area**				< 0.001
Medicine	5,687	11.4	10.6–12.3	
Surgery	3,573	10.3	9.3–11.3	
Gyn/Obs	687	2.6	1.6–4.1	
ICUs	614	22.0	18.8–25.5	
Paediatrics	581	1.6	0.7–2.9	
Rehabilitations	492	11.2	8.5–14.3	
Psychiatry	381	0.8	0.2–2.3	
Others	148	8.11	4.3–13.7	
Neonatology	146	0	0-2.5	
Combinations	92	9.8	4.6–17.8	
Long term care	5	40.0	5.3–85.3	

Among the 1,385 HAIs reported, the most common types were bloodstream infections (BSI, 262 cases, 18.9%), followed by urinary tract infections (UTI, 237 cases, 17.1%), SARS-CoV-2 infections (236 cases, 17.0%), pneumonia and lower respiratory tract infections (PN-LRTI, 231 cases, 16.7%), surgical site infections (SSI, 152 cases, 11.0%), and gastrointestinal tract infections (GI, 103 cases, 7.4%). See Table 2 for complete results.

**Table 2:** Types of HAI

Types of HAIs	Frequency *N*. (%)	Prevalence (%)
Bloodstream infections	262 (18.9)	2.1
Urinary tract infections	237 (17.1)	1.9
SARS-CoV2 infection	236 (17.0)	1.9
Pneumonia and lower respiratory tract infections	231 (16.7)	1.9
Surgical site infections	152 (11.0)	1.2
Gastrointestinal tract infections	103 (7.4)	< 1
Systemic infection	45 (3.2)	< 1
Skin and soft tissue infection	34 (2.5)	< 1
CVC or PVC-related local/systemic infections	25 (1.8)	< 1
Bones and joints infections	19 (1.4)	< 1
Cardiovascular system infections	15 (1.1)	< 1
Central nervous system infections	10 (< 1)	< 1
Reproductive system infections	8 (< 1)	< 1
Infections of the eye, ear nose or oral cavity	8 (< 1)	< 1

### Isolated microorganisms

**Fig. 1:**
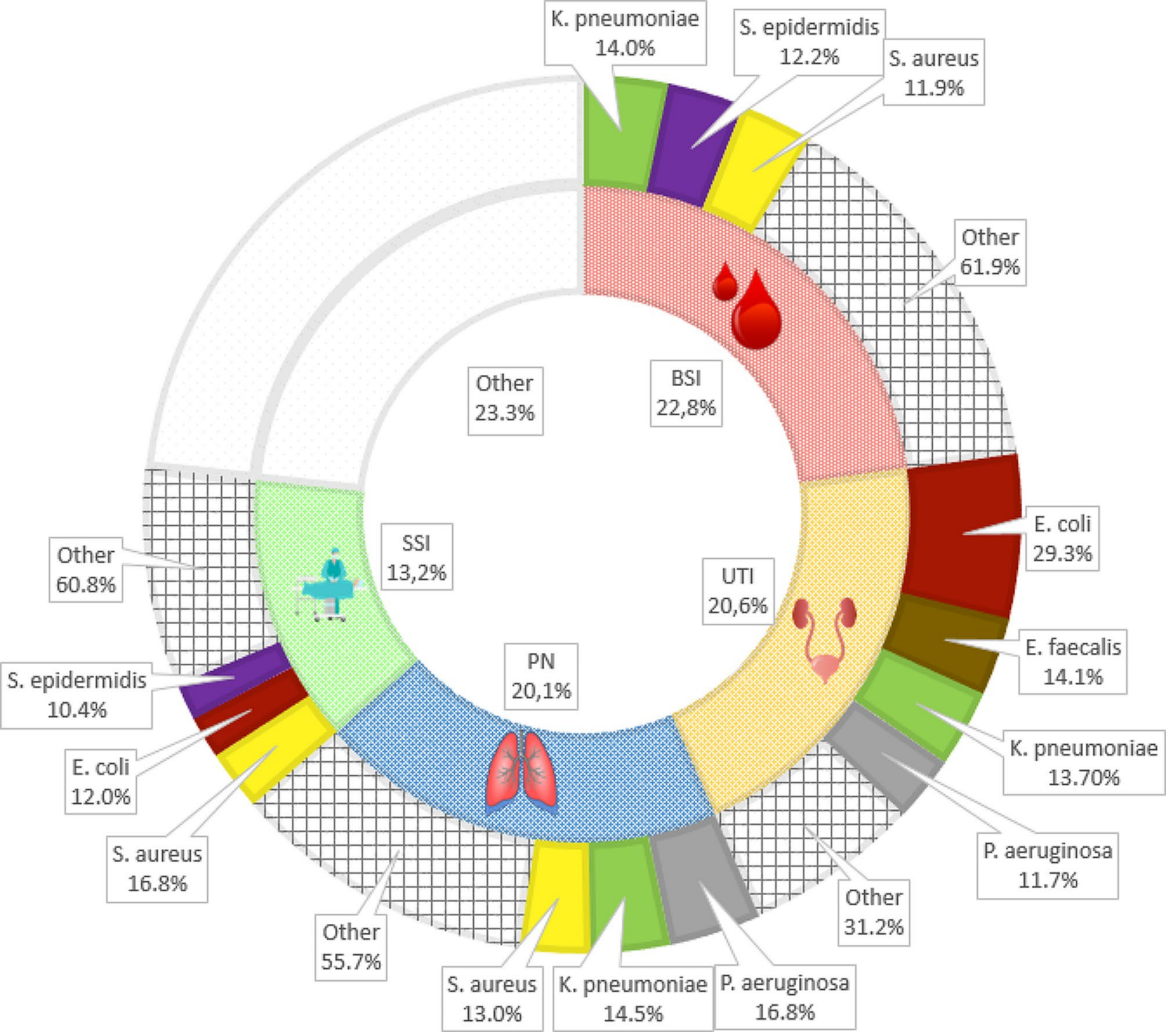
Distribution of HAIs per site and most frequently isolated microorganisms

Laboratory detection was achieved for 887 HAIs (64% of total HAIs), with 1,039 microorganisms isolated (up to 2 microorganisms per HAI). A total of 71 different pathogens were identified. The most frequently isolated microorganisms included SARS-CoV-2 (145, 14%), *E. coli* (128, 12.3%), *K. pneumoniae* (108, 10.4%), *S. aureus* (94, 9%), and *P. aeruginosa* (77, 7.4%). Figure 1 shows the most frequently isolated microorganisms per HAI type.

Regarding antibiotic resistance, *S. aureus* was resistant to oxacillin in 35.3% (*n* = 30) of cases; *K. pneumoniae* was resistant to third generation cephalosporins in 53.4% (*n* = 55) of cases and in 21.8% (*n* = 22) of cases to carbapenems; *P. aeruginosa* and *A. baumannii* were respectively resistant to carbapenems in 24.2% (*n* = 16) and 89% (*n* = 8) of cases.

There were also 4 confirmed cases (0.6%) and 1 possible case (0.1%) of pan-drug-resistant microorganisms, meaning they were resistant to all tested antibiotics. These cases included two *A. baumannii*, one *K. pneumoniae*, and one *P. aeruginosa*, with one possible case of *K. pneumoniae*.

### Antimicrobial use

5,225 patients (42.1%, 95% CI 41.3–43.0%) were on systemic antimicrobial therapy. The total number of antimicrobial therapies was 6,884, with each patient receiving 1.32 medications on average. In Table 3, we provide data regarding antimicrobial use stratified by area of care (top 5), most frequently used molecule (top 5), antimicrobial class (top 5), AWaRe classification and clinical indication. The prevalence of patients on therapy varied significantly across the areas, with the highest prevalence registered in ICUs. The most used antimicrobials were Piperacillin associated with enzyme inhibitors and Ceftriaxone. According to the WHO AWaRe classification [21, 22], the most used antibiotics belonged to the Watch class, followed by the Access class and the Reserve class; for 300 antimicrobials the AWaRe classification was not applicable (antifungal and antituberculosis drugs). Antimicrobials were mostly used to treat community-acquired infections followed by HAIs.

**Table 3:** Antimicrobial use stratified by area of care (top 5), most frequently used molecule (top 5), antimicrobial class (top 5), AWaRe classification and clinical indication

Antimicrobial Use	
**Area of care (top 5)**	**Prevalence % (95% C.I.)**
Intensive care unit	50.3% (46.4–54.5)
Internal medicine	47.4% (46.1–48.8)
General surgery	47.1% (45.5–48.8)
Gynecology	30.9% (27.4–34.4)
Pediatrics	28.6% (24.9–32.4)
**Antimicrobial molecule (top 5)**	***N (%)***
Piperacillin associated with enzyme inhibitors	1,016 (14.8%)
Ceftriaxone	989 (14.8%)
Cefazolin	674 (9.8%)
Meropenem	413 (6.0%)
Amoxicillin associated with enzyme inhibitors	369 (5.4%)
**Antimicrobial class (top 5)**	***N (%)***
Cephalosporins	2043 (29.7%)
Penicillins Carbapenems	1,896 (27.5%) 468 (6.8%)
Quinolones Glycopeptides	368 (5.3%) 319 (4.6%)
**Antimicrobial AWaRe classification**	***N (%)***
Watch	3,926 (57%)
Access	2251 (32.7%)
Reserve	407 (5.9%)
Not applicable	300 (4.4%)
**Clinical indication**	***N (%)***
Community-acquired infections	2,882 (41.9%)
Healthcare-associated infections	1339 (19.5%)
Surgical prophylaxis	1,163 (16.9%)
Medical prophylaxis	812 (11.8%)
Long-term-care-associated infections	86 (1.2%)
Unknown/not reported	602 (8.7%)

Upon further analysis, antibiotics used for treating infections (including community-acquired, healthcare-associated, and long-term-care-associated) mostly belonged to the Watch class (2,906, 71.8%), followed by the Access class (792, 19.6%) and the Reserve class (349, 8.6%).

Antimicrobials used to treat infections were mostly used to treat pneumonia (1,301, 31.4%), followed by bacteremia with laboratory confirmation (370, 8.9%), lower-urinary-tract infections (362, 8.7%) and intra-abdominal sepsis (332, 8.0%).

Antibiotics used for prophylaxis mostly belonged to the Access class (1,267, 67.4%), followed by the Watch class (595, 31.6%) and the Reserve class (18, 1.0%).

**Fig. 2:**
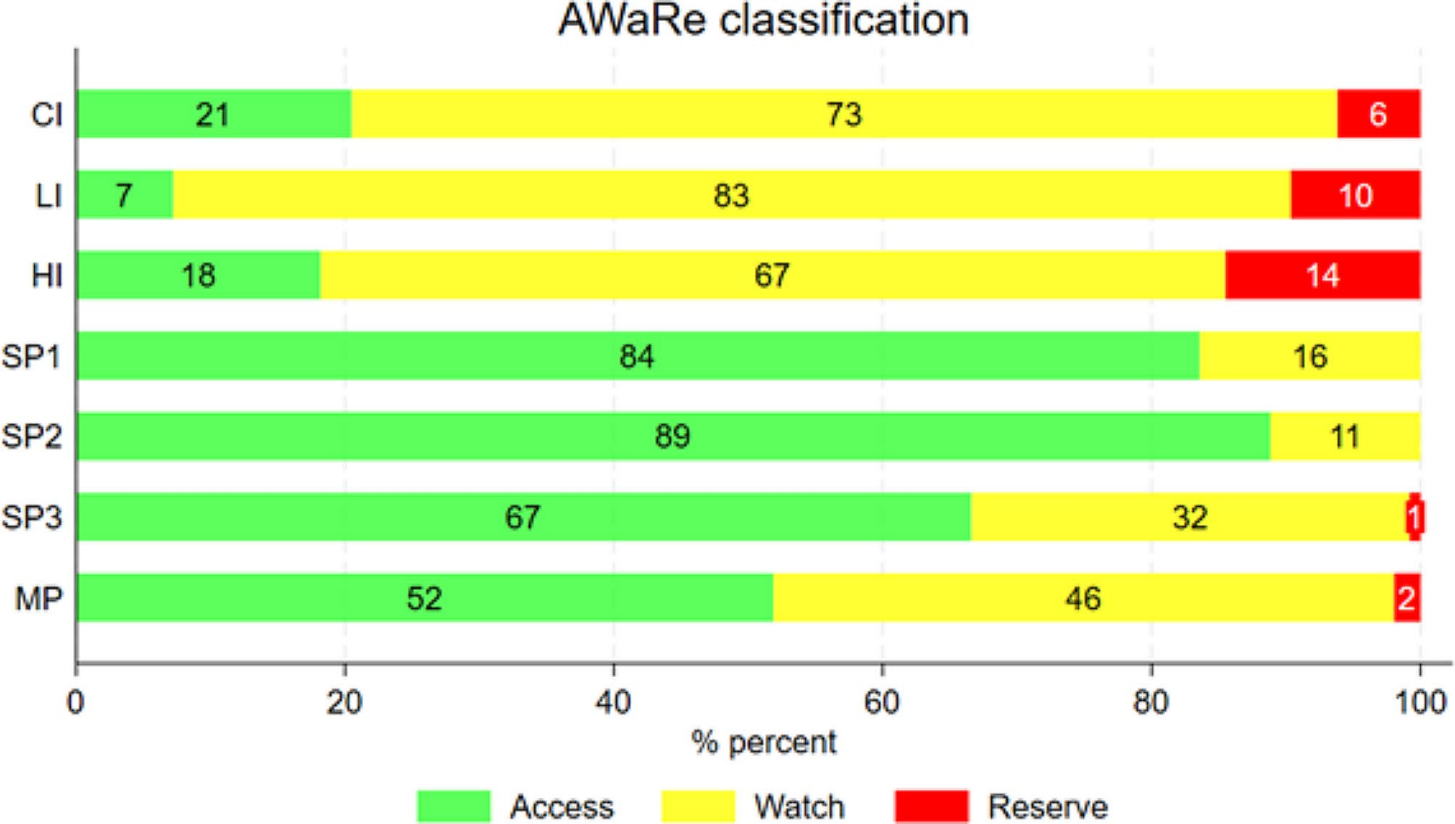
Distribution of the AWaRe classification for each antibiotic indication Legend: CI: community-acquired infections; LI: long-term acquired infections; HI: Healthcare-associated infections; SP1: Surgical prophylaxis (single dose); SP2: Surgical prophylaxis (1 day); SP3: Surgical prophylaxis (> 1 day); MP: Medical prophylaxis

Antimicrobials used for surgical prophylaxis lasting more than one day (43% of all surgical prophylaxis), compared to prophylaxis lasting one day or less belonged significantly more to the Watch class (32.3% vs. 14.9%, *p* < 0.001) and significantly less to the Access class (66.1% vs. 84.8%, *p* < 0.001). Figure 2 summarize the distribution of the AWaRe classification for each antibiotic indication.

## Discussion

In 2022 Lombardy, the most populated Italian region, participated in the PPS-3 collecting data from over 12,000 patients. The overall prevalence of HAIs, excluding healthcare-associated COVID-19, registered in Lombardy (8,4%) reveals an upward trend when compared to the preceding national survey (8.0%) [23] and surpasses the latest available European average (6.5%) [10].

Among the four most frequent infections (75% of all HAIs), it is noteworthy the significant rise in BSI, twice the European rate in 2016-17 and surpassing Italian figures from 2016 [23, 24].

Furthermore, the study has shown a higher prevalence of HAIs in the ≥ 65 age group with a total prevalence increasing up to 12.5%; in groups with a more severe McCabe score due to the frailty of the patients; in intensive care units (22%), with twice the value of the medical wards (11.4%), rehabilitation units (11.2%) and surgical departments (10.3%); in the presence of invasive MDs with a prevalence of 31.1% in intubated patients, 24.8% in patients with central venous catheter and 17.2% in patients with a urinary catheter.

Of the 12,412 patients, 42.1% were on antimicrobial therapy, consistent with the previously recorded Italian data (44.5%) [23], but higher than the European data (30.5%) [25].

In terms of antibiotic class selection, according to the World Health Organization’s AWaRe (Access-Watch-Reserve) classification [21, 22], results are not reassuring: the Access group (antibiotics less likely to induce resistance), the Watch group (broader spectrum and need for restricted use) and the Reserve group (last resort indication) account respectively for 32.7%, 57% and 5.9% of total antibiotic consumption. While the general WHO target for Access antibiotics is set at 60%, it is worth noting that there are no specific targets outlined for hospitals: our data align with ECDC’s data which shows for 2022 an average usage of 5.2% for the Reserve group (the sole category represented for hospital use) in Europe and 7.8% in Italy [26].

Regarding the indication, medical prophylaxis (11.9%) showed a significant reduction compared to the 23.3% observed in the Italian PPS-2 [23]; however, it still represents a high percentage, at least in part due to misclassification in the collection of data owing to the circumstance that data collectors could have reported erroneously empirical therapies as medical prophylaxes. The use for surgical prophylaxis is significant (17.1%) and particularly critical when its duration exceeds one day (43% of surgical prophylaxis), an unjustified use, and more likely to be carried out using an antibiotic from the Watch class, resulting in a dual error.

From a microbiological perspective, investigations were conducted only on 64% of the total HAIs, however they showed a significant pattern of antibiotic resistance. Focusing on antibiotic resistance data of the Italian PPS-2 [23] and the results of our study, we observed a lower level of antimicrobial resistance of *S. aureus* to oxacillin (47.4% and 35.3%), of *K. pneumoniae* to third-generation cephalosporins (68.1% and 53.4%) and to carbapenems (49.5% and 21.8%), and of *P. aeruginosa* to carbapenems (31% and 24.2%). Notably, antimicrobial resistance of *A. baumannii* to carbapenems was higher (76.9% and 89%). It should be noted, however, that the data were collected based on information provided by ACHs, and there may have been instances where pathogen isolation was assigned without a comprehensive evaluation of the precise infection aetiology.

### Strengths and limitations of the study

There are some limitations in this study, predominantly stemming from the extensive nature of large multicenter surveys. We have to take into account that, despite healthcare professionals collecting data using a standardized definition of HAI, errors and misinterpretations among data collectors may have occurred. To address this issue, an initial coherence analysis was conducted and is provided in Appendix 1. However, these measures might not be sufficient, particularly given the absence of an external validation process, as recommended by the ECDC protocol [17].

Furthermore, it is important to note that the prevalence of infections differs from the incidence, as data collected on a single day may not be representative of reality, especially for hospitals with fewer patients. As demonstrated by Gastmeier et al., prevalence studies tend to show a higher rate of infection compared to incidence rate studies [27], but incidence studies are costly, time-consuming and require many resources, making it difficult to involve a large number of hospitals as effectively as in prevalence studies, complicating the gathering and comprehensive comparison of results.

Additionally, the healthcare system in Lombardy is constituted of highly specialized hospitals that attract patients from across the country, leading to higher complexity of cases which, in turn, increases the likelihood of admitting patients with greater frailty compared to other regional contexts, potentially resulting in a more significant impact of HAIs.

Despite these limitations, the data analyzed in this study offer a valuable contribution to understanding the impact of HAIs on patients admitted to ACHs in Lombardy and can be used as a baseline indicator for future comparisons.

### Implications for policy and practice

The first two editions of the Point Prevalence Survey (PPS), conducted in 2011 and 2016, paved the way for establishing a surveillance system for healthcare-associated infections (HAI) and antibiotic use at both the European and national levels. This was achieved through the structuring of extensive databases capable of supporting targeted analyses that can facilitate interventions aimed at improving the quality of care provided. The Lombardy Region participated in both previous editions, despite the participation of few hospitals. However, in the third prevalence study, there was a significant increase in participation which has been crucial in developing the first regional report. The analysis of the collected data will help identify common challenges, provide new tools to promote and strengthen the understanding of phenomena, enhance the skills of all stakeholders, and offer recommendations and strategies for managing HAIs and the conscious use of antibiotics. Furthermore, it represents an important regional benchmarking for internal analysis that every ACH is called upon to conduct to establish concrete improvement objectives.

## Conclusions

Healthcare-Associated Infections pose a significant concern in the current healthcare setting, with substantial implications for both patients and ACHs. PPS-3 in Lombardy facilitated the collection of data on HAIs from ACHs, providing a structured benchmarking framework to guide regional health policies and reduce the burden of HAIs. Along with prevention activities and prudent use of antimicrobials, surveillance protocols of HAIs, like the ECDC Point Prevalence Survey, must be adopted at all healthcare institutional levels (hospital, regional, national, international) as they are an indispensable source of data for the implementation of routinary and extraordinary initiatives for the prevention and control of HAIs in the antimicrobial resistance era.

### Electronic supplementary material

Below is the link to the electronic supplementary material.

Supplementary Material 1: Coherence analysis. Coherence analysis identifying records with logical inconsistencies and corrective actions taken for each.

## Data Availability

The datasets generated and/or analyzed during the current study are not publicly available. All Lombardy hospital data are accessible solely to the regional coordinator in accordance with the privacy rules outlined by the PPS protocol. However, they can be made available from the corresponding author upon reasonable request, upon authorization from the regional coordinator.

## Abbreviations

HAI: Healthcare-Associated Infection
ECDC: European Centre for Disease Prevention and Control
PPS-3: Point Prevalence Survey-3
SARS-CoV-2: Severe Acute Respiratory Syndrome coronavirus-2
ICU: Intensive Care Unit
WHO: World Health Organization
AWaRe: Access, Watch and Reserve
EU/EEA: EEA, European Union/ European Economic Area
UK: United Kingdom
ACH: Acute Care Hospital
GDPR: General Data Protection Regulation
SSI: Surgical Site Infections
CI: Confidence Interval
IQR: Interquartile Range
MD: Medical Device
BSI: Bloodstream Infections
UTI: Urinary Tract Infections
PN-LRTI: Pneumonia and Lower Respiratory Tract Infections
GI: Gastrointestinal tract Infections
