# Supplementary material for: Healthcare-associated infections and antimicrobial use in acute care hospitals: a point prevalence survey in Lombardy, Italy, in 2022
Source: BMC Infect Dis. 2024 Jun 25;24:632. doi: 10.1186/s12879-024-09487-7 (PMC11197227; doi:10.1186/s12879-024-09487-7)
Supplement: Supplementary file 1 — Supplementary Material 1: Coherence analysis. Coherence analysis identifying records with logical inconsistencies and corrective actions taken for each. [file 12879_2024_9487_MOESM1_ESM.pdf]

| FLAG | DEFINITION | DESCRIPTION | ERRORS DETECTED (N.) | CORRECTIVE ACTIONS ON DATABASE | CONSIDERATIONS FOR DATA ANALYSIS | NOTES |
|---|---|---|---|---|---|---|
| FLAG 0 | Patient survey date ≠ Ward survey date | Survey date on form A (patient data) is not consistent with survey date on other forms A of the same ward | 107 | Survey date corrected by replacing it with the date present on the other forms A assignable to the same ward, if uniquely identifiable | | |
| FLAG 1 | Survey date < Date of hospital admission | Survey date precedes the date of hospital admission | 60 | For N=1 record: replaced the hospital admission date with the date of onset of HAI marked as present at hospital admission | If the hospital admission date is incorrect (N=59) records not considered in calculations where the admission date is relevant | |
| FLAG 2 | Survey date < date_hai1 | The survey date precedes the onset date of HAI | 6 | For N=1 record: corrected the onset date of HAI to have a consistent month | If the onset date of HAI is incorrect (N=5) records not considered in calculations where the onset date of HAI is relevant | |
| FLAG 3 | Survey date < date_hai2 | The survey date precedes the onset date of HAI | 2 | For N=2 records: corrected the onset dates of HAI to have a consistent month | | |
| FLAG 4 | Survey date < November 1, 2022 or > November 30, 2022 | The survey was not conducted in November 2022 | 93 | | | For N=31 records: survey date December 2, 2022; For N=62 records: survey date December 20, 2022 |
| FLAG 5 | Indication_antimicrobial1=HI and hai_patient=NO | The patient does not have HAI but is prescribed an antibiotic indicated for HAI treatment | 59 | | No corrective action taken: presumably antibiotic for a cured HAI | |
| FLAG 6 | Indication_antimicrobial2=HI and hai_patient=NO | The patient does not have HAI but is prescribed an antibiotic indicated for HAI treatment | 19 | | No corrective action taken: presumably antibiotic for a cured HAI | |
| FLAG 7 | Indication_antimicrobial3=HI and hai_patient=NO | The patient does not have HAI but is prescribed an antibiotic indicated for HAI treatment | 3 | | No corrective action taken: presumably antibiotic for a cured HAI | |
| FLAG 8 | Hai_patient=YES, code_hai1 filled and code_hai2=empty but microorganism reported both in hai1 and hai2 microrganism section | The patient has HAI, the HAI code 2 is empty, but the microorganisms associated with HAI 2 are filled in. | 6 | The microorganism codes for HAI 2 have been deleted as it was not possible to determine whether the intention was to mark another HAI on the same patient or to add an additional microorganism to the first HAI, potentially exceeding the limit of two microorganisms per HAI | | |
| FLAG 9 | Hai_patient=YES (HAI ≠ COVID-19), but antimicrobial_patient=NO (no antibiotics administered) | Patient has HAI (non-COVID) but is not taking any antibiotics | 45 | | No corrective action taken: HAI without therapy | |

| | | | | | | |
|---|---|---|---|---|---|---|
| FLAG 10 | Hai_patient=YES, but code_hai1/2 = empty | Patient has HAI, but the type of HAI has not been specified | 20 | If it was not possible to deduce the type of HAI from other fields due to inconsistency: the patient was considered as not having HAI (N=5). If it was possible to deduce the type of HAI from other fields, a compatible code was inserted (N=15): this latter operation did not modify the prevalence of HAIs but allowed for an estimate of the type of HAIs consistent with the prevalence of HAIs | | |
| FLAG 11 | Presence_hai1=YES, date_hai1 ≠ empty and > date_hospital_admission | HAI present on the time of hospital admission but onset date of HAI indicated as occurring after hospital admission | 49 | Removed the onset date of HAI in all cases where HAI was present on hospital admission | | According to protocol, the field for the onset date of HAI should not be filled if HAI is present upon hospital admission |
| FLAG 12 | Presence_hai2=YES, date_hai2 ≠ empty and > date_hospitaladmission | HAI present at the time of hospital admission but onset date of HAI indicated as occurring after hospital admission | 6 | Removed the onset date of HAI in all cases where HAI was present on hospital admission | | According to protocol, the field for the onset date of HAI should not be filled if HAI is present upon hospital admission |
| FLAG 13 | Patient age in months > 24 | Age expressed in years indicated in the "Age expressed in months" column | 18 | Age considered correct in years and moved to the "Age in years" column | | |
